# Tricuspid regurgitation, right ventricular function, and renal congestion: a cardiorenal triangle

**DOI:** 10.3389/fcvm.2023.1255503

**Published:** 2023-10-04

**Authors:** Ilana Forado-Benatar, Pedro Caravaca-Pérez, Diana Rodriguez-Espinosa, Joan Guzman-Bofarull, Elena Cuadrado-Payán, Yasbanoo Moayedi, José Jesús Broseta, Marta Farrero

**Affiliations:** ^1^ Department of Cardiology, Hospital Clínic of Barcelona, Barcelona, Spain; ^2^ Department of Nephrology and Renal Transplantation, Hospital Clínic of Barcelona, Barcelona, Spain; ^3^ Ted Rogers Centre for Heart Research, Peter Munk Cardiac Centre, University Health Network, Toronto, ON, Canada

**Keywords:** tricuspid regurgitation, right ventricular function, heart failure, abdominal congestion, cardiorenal, diuretic resistance, ultrafiltration

## Abstract

There is a growing interest in the evaluation of tricuspid regurgitation due to its increasing prevalence and detrimental impact on clinical outcomes. Historically, it has been coined the “forgotten” defect in the field of valvular heart disease due to the lack of effective treatments to improve prognosis. However, the development of percutaneous treatment techniques has led to a new era in its management, with promising results and diminished complication risk. In spite of these advances, a comprehensive exploration of the pathophysiological mechanisms is essential to establish clear indications and optimal timing for medical and percutaneous intervention. This review will address the most important aspects related to the diagnosis, pathophysiology and treatment of tricuspid regurgitation from a cardiorenal perspective, with a special emphasis on the interaction between right ventricular dysfunction and the development of hepatorenal congestion.

## 1. Introduction

Tricuspid regurgitation (TR) is a common condition most commonly associated with degenerative and left-sided heart disease (1, 2). Right-sided cardiac chambers and TR have been historically overlooked and mostly become relevant during the surgical evaluation of left valvular heart disease.

TR can lead to dysfunction of the right ventricle (RV) due to increased preload and progressive remodeling. This consequently results in signs and symptoms of heart failure (HF) and subsequently contributes to a spectrum of liver and kidney impairment (3–5) primarily attributable to persistent right-sided congestion. The interplay between the cardiovascular and renal systems becomes evident in the context of cardiorenal syndrome, wherein chronic abnormalities in heart function leads to kidney dysfunction (6).

TR has long been considered a relatively benign entity resulting in the underutilization of diagnostic and therapeutic resources. The underestimation of the clinical impact of TR has led to a pattern of care delaying intervention until very advanced stages of disease where the risks of treatment become excessively elevated (7). While surgical treatment of TR, even in early stages, has been associated with poor outcomes, innovative percutaneous techniques have emerged as a potential alternative. These percutaneous techniques have expanded their scope beyond the conventional surgical indications, encompassing high-risk patients previously excluded from surgical studies (8). In addition to mechanical treatments decongestion management in TR is of paramount importance, so the cardiorenal interaction is a key factor. Hemodynamic profiling of patients and the assessment of right ventricular function and systemic congestion are crucial to determine optimal timing and indications for percutaneous interventions along with identifying who would not derive any benefit from further interventions.

## 2. Tricuspid regurgitation: prevalence, prognosis, and diagnosis

TR is frequently encountered in the echocardiography laboratory. While mild TR may not have significant consequences, advanced degrees of TR are associated with factors such as advanced age, female sex, and left sided heart disease. Moderate to severe TR is present in 4%–6.6% of patients older than 75 years and is associated with increased mortality and morbidity independent of RV dysfunction and pulmonary hypertension (1, 2).

Primary TR is the least prevalent etiology, representing only 8%–10% of all TR cases and is mainly related to pathologies affecting the tricuspid apparatus (papillary muscles, chordae and annulus) and tricuspid valve (TV) leaflets. Causes of primary TR include carcinoid syndrome, rheumatic heart disease, congenital abnormality of TV, iatrogenic valve damage and endocarditis. Lead impingements from cardiac implantable electronic devices are a leading cause of primary TR, as it has been reported to occur in up to 38% of patients following implant (9–11).

Secondary TR can arise as a consequence of various factors, including RV dilatation due to volume overload or increased pulmonary pressures. It may also result from dilatation of the right atrium or tricuspid annulus as occurs in atrial fibrillation. Additionally left heart or valvular diseases can contribute to the development of secondary TR. Interestingly, 8% of cases of secondary TR can occur in isolation (1). In the setting of left-sided heart pathology, TR is a late event indicating a more advanced disease, higher pulmonary pressures, extensive ventricular remodeling, and worse prognosis (12). Without appropriate intervention, TR perpetuates a vicious pathophysiological cycle that leads to further adverse remodeling of the ventricles, atria, tricuspid annulus, with increasing TR severity and subsequent irreversible RV dysfunction leading to unfavorable outcomes (7, 13).

Transthoracic echocardiography (TTE) is the preferred diagnostic method for TR, allowing TV visualization as well as RV function (10). Despite its widespread use, TTE has limitations in TV assessment, since only two leaflets can be visualized simultaneously. 3D echocardiography, an extension of TTE, provides simultaneous visualization of the three leaflets and surrounding structures including a complete visualization of the annulus, providing a better characterization of TV. The addition of color flow Doppler allows to quantify the severity of TR by analyzing the effective regurgitant orifice size, regurgitant volume, the TR jet, and the vena contracta width. Of note, transesophageal echocardiography (TEE) is no more effective in providing optimal views of the TV than TTE (10, 14).

## 3. Tricuspid regurgitation and right ventricle

The impact of TR on the RV varies on a number of factors including the underlying cause, degree of anatomical alteration, presence of pulmonary hypertension, left valvular heart disease or presence of atrial fibrillation (AF) (15, 16). Several phenotypical classifications have been used to predict outcomes. Deitz et al. describe four RV remodeling patterns based on RV dilatation and dysfunction in patients with secondary moderate to severe TR: (1) normal RV size with normal function, (2) dilated RV with normal function, (3) normal RV size with reduced function, and (4) dilated RV with reduced function. The 4th pattern is common in patients with severe TR, accounting for 43%. Any degree of RV dysfunction (patterns 3 and 4) is associated with decreased survival when compared to patients with normal RV function (17). Two additional phenotypes of functional TR have been described: atrial functional TR (A-FTR) and ventricular functional TR (V-FTR). A-FTR refers to TR associated with AF leading to a progressive dilatation and enlargement of the tricuspid annulus associated with right atrial enlargement (18, 19). In this group, the RV changes its shape into a conical remodeling pattern with annular enlargement and dilatation of the RV basal segments despite a normal RV length (20). In contrast, in V-FTR [as seen in left-sided heart disease, RV dysfunction or pulmonary hypertension (PH)], the RV takes on a spherical or elliptical pattern as a consequence of a longitudinal remodeling with valvular tethering, while tricuspid annulus enlargement is only mild (18, 19, 21, 22). Patients with V-FTR have worse survival compared with patients with A-FTR, especially in the PH subgroup (23). RV function is the main prognostic factor in HF and in TR. Therefore, its evaluation is essential for risk stratification (7, 24).

As in the case of TR, TTE remains the primary imaging modality for RV assessment, for practical reasons. Conventional TTE parameters such as tricuspid annular systolic excursion (TAPSE), tissue Doppler velocity at the lateral tricuspid annulus and fractional area change rely on geometric assumptions and are load and angle-dependent leading to an overestimation of RV function in severe TR (25). Other parameters, such as free wall longitudinal strain, can provide a more accurate assessment of RV function (26). TAPSE/SPAP (systolic pulmonary arterial pressure), a parameter of ventricular arterial coupling, which assesses the relationship between RV contractility and afterload, has proven to be a valuable prognostic parameter in this context (27, 28). In general, multimodality imaging would be the preferred approach for RV assessment in the context of severe TR, with cardiac magnetic resonance as the gold standard for evaluating RV volumes and function (29–31).

## 4. Right ventricular failure and abdominal congestion: renal, hepatic and intestinal consequences

TR causes an elevation of RV preload leading to a decrease in cardiac output, and an increase in venous congestion. Consequently, signs and symptoms of right heart failure (RHF) include an elevated jugular venous pressure, edema, ascites, hepatomegaly and, in advanced stages, renal and hepatic failure with malnutrition (32). Congestion is one of the main manifestations of RV dysfunction and it is associated with worse quality of life, frequent admissions, and increased mortality. As the disease progresses, a shift in the clinical phenotype can be seen: with a low cardiac output state can advance towards a high cardiac output state secondary to hepatic failure and portal hypertension. Decreased splanchnic wash-out of vasoactive substances often leads to vasoplegia and high and a hyperdynamic state resulting in hepatorenal failure. End stage RV failure is associated with a poor response to percutaneous treatment of TR (33).

### 4.1. Congestive nephropathy

Observational studies have consistently highlighted the strong association between TR and renal dysfunction. These studies have underscored the adverse effects of TR on clinical outcomes. In fact, less than 18% of the patients with functional TR have a normal renal function, defined by an eGFR greater than 90 ml/min/1.73 m^2^ (5). The persistent elevation of central venous pressure is the main determinant of kidney dysfunction.

The glomerulus has the remarkable ability to maintain relatively stable glomerular filtration despite reduced renal plasma flow by regulating the vascular tone of intraglomerular vessels. Activation of the renin angiotensin system causes vasoconstriction predominantly of the efferent arteriole, increasing intraglomerular pressure and filtration fraction (5, 34, 35). Severe TR leads to renal venous hypertension in peritubular, interstitium and tubular lumen which counteracts intraglomerular pressure and deteriorates GFR (glomerular filtration rate).

Another critical aspect of kidney damage is the presence of diuretic resistance related to the presence of tubular dysfunction and increased sodium avidity (36). Neurohormonal activation and hemodynamics perturbance increase active sodium and water reabsorption in the renal tubules, perpetuating congestion and a diminished diuretic response (5, 34).

### 4.2. Congestive hepatopathy

Passive chronic congestion from TR leads to an increase in hepatic venous pressure and, thus, to sinusoidal hypertension. Over time, this causes hepatocyte atrophy and apoptosis, sinusoidal thrombosis and centrilobular fibrosis, which can eventually lead to cardiac cirrhosis. The coexistence of reduced cardiac output and decreased hepatic perfusion may aggravate the problem. Liver damage caused by congestion may be subclinical with primary evidence from alterations in hepatocellular laboratory tests including mild hyperbilirubinemia and elevation of cholestasis markers (alkaline phosphatase and gamma-glutamyl transferase). If liver damage progresses, increased serum aminotransferase, hypoalbuminemia and coagulopathy can be seen. Liver disease can become as clinically relevant with progressive cardiac disease which further complicates its management (37–39).

### 4.3. Intestinal congestion

The relationship between HF and the gastrointestinal system is complex and not yet fully understood. RV dysfunction has been linked with malabsorption, loss of appetite and weight loss, often culminating in the development of cardiac cachexia. Splanchnic congestion, a hallmark of RV dysfunction, exerts changes on gut morphology, permeability, and function. These alterations can result in changes in the composition of the gut microbiota, potentially impacting processes like bacterial translocation and activation of inflammatory pathways (40).

## 5. Multi-parametric approach to congestion in TR

When evaluating congestion in TR, it is crucial to employ diverse diagnostic methods. These may encompass clinical scoring systems, imaging techniques, bioimpedance measurements, hemodynamic tests, and analysis of circulating biomarkers.

### 5.1. Venous excess ultrasound score (VExUS)

The Venous Excess Ultrasound Score (VExUS) is a valuable tool for assessing a patient's volume status through point-of-care ultrasound (41, 42). The scoring system assesses congestion by evaluating the diameter of the inferior vena cava (IVC) and the Doppler waveform patterns of hepatic, portal, and intrarenal veins. VExUS offers a practical and effective means of gauging congestion in a clinical setting (43).

While ultrasound volume assessment is at times considered less informative in patients with TR due to its association with elevated right atrial pressure, it is important to recognize that patients with TR can still experience clinical overload, even without absolute excess volume. Therefore, severity indicators identified through ultrasound warrant careful consideration, especially those unaffected by right atrial pressure, such as the Doppler portal vein pulsatility index and the intrarenal veins waveform, as they reflect right-sided heart hemodynamics. It is worth noting that while portal Doppler remains unaffected by right atrial pressure, a false negative interpretation can occur in the setting of liver cirrhosis due to centrilobular fibrosis. Therefore, a scenario of a seemingly normal pattern (pulsatility <30%) can mask hepatic congestion (44). In TR, key intrarenal waveform findings include an elevated x-descent and a significant increase in the v-wave.

### 5.2. Biomarkers

In patients with right heart failure (RHF), particularly those with significant TR, natriuretic peptides may not provide additional prognostic information as their levels tend to be lower than in patients with reduced left ventricular ejection fraction. Alternatively, CA-125, a glycoprotein synthetized by serous epithelial cells, has been found to better reflect right heart function and may have a stronger predictive value in RV failure and venous congestion. CA-125 concentrations are not influenced by factors like renal function, age, or weight (45–47). Another biomarker, bio-adrenomedullin (bioADM), has been shown to have an association with various signs of right-sided cardiac dysfunction such as peripheral edema, hepatomegaly, and ascites (48). Soluble ST2 has been linked to echocardiographic signs of RHF and central venous pressure, and it can serve as an indicator of diuretic resistance (49, 50). Urinary albumin level is a reliable indicator of the severity of RHF, with higher levels associated with a more advanced functional class and symptoms of congestion. Moreover, urinary albumin has been closely associated with other congestion biomarkers (51). Another intriguing urinary biomarker is sodium excretion:low levels of natriuresis following a furosemide stress test suggest a greater likelihood of inadequate diuretic response and improper decongestion (52).

## 6. Treatment strategies

### 6.1. Congestion treatment in RV failure

The evidence for guideline-directed medical therapy for TR is lacking. There is a limited armentarium in treatment options beyond addressing the underlying causes of secondary tricuspid regurgitation and diuretics (Class IIa indication in the 2022 European guidelines) (53). Loop diuretics, such as furosemide, torasemide, and bumetanide, are commonly used, with varying oral bioavailability and efficacy in patients with RHF. When loop diuretics are insufficient, a sequential nephron tubular blockade treatment may be necessary, combining thiazides, acetazolamide, iSGLT2 or even vaptanes. A more in-deep review in that sense has been previously published by our group (54). In cases of refractory congestion secondary to severe TR, the diuretic response can be impaired both in acute and chronic phases. Therefore, extracorporeal ultrafiltration may need to be considered. Hemodialysis, hemodiafiltration and ultrafiltration can be used according to the specific situation. Of note, in some severe cases of TR and refractory congestion, peritoneal dialysis (PD) has been shown to be beneficial as a chronic treatment, even in patients with mildly impaired glomerular filtrate, as strategy to optimize RV filling pressures, resulting in an improvement in quality of life, reduced hospitalizations, and enhanced renal and cardiac function (55–60).

### 6.2. Inotropes and other cardiovascular drugs

In contrast to left-sided HF, the evidence supporting the use of standard HF medications other than diuretics for the right side is not well established and may vary according to the underlying cause and the acute or chronic presentation. Inotropes can be used to support RV function and optimize left ventricular filling pressures. Levosimendan and milrinone may be of particular interest due to their pulmonary vasodilatation properties, that would diminish RV afterload (61), but careful monitoring of systemic perfusion pressures needs to be considered. Digoxin may also be beneficial considering its potential inotropic effect over the RV, although the available evidence in this specific situation is limited and heterogeneous, with no conclusive data on potential improvements in RV ejection fraction or NYHA class (62). In the case of RV failure with normal left ejection fraction, beta-blockers may be deleterious due to their negative inotropic and chronotropic effect (63, 64).

### 6.3. Percutaneous treatment for TR: techniques and clinical implications

Isolated surgery for severe TR has a class IIa indication in symptomatic patients, however, it is limited by its high in-hospital mortality rate. As an alternative, percutaneous interventions have emerged for inoperable or very high-risk patients, with a Class IIb indication in the recent European guidelines (53).

Percutaneous intervention for TR includes a range of devices:

1) Coaptation devices: these include leaflet approximation with tricuspid transcatheter edge-to-edge repair (T-TEER) or spacers devices, whose objective is to increase leaflet coaptation. Initial experience with T-TEER systems have shown potential benefits in observational trials, including a decrease in hospitalizations for HF (65), increase in cardiac output, improved liver enzymes (66), reverse RV remodeling (67) and improved nutritional status (32). The recently published TRILUMINATE trial, randomly assigned 350 symptomatic severe TR patients to T-TEER or placebo, showing an improvement in quality-of-life questionnaires with no differences in mortality or HF hospitalization rates (68).
2) Percutaneous annuloplasty: this involves procedures to reduce the diameter of the tricuspid annulus. The Cardioband (Edwards Lifesciences, Irvine, CA, USA) system facilitates a percutaneous annuloplasty, reducing the diameter of the tricuspid anulus to facilitate leaflet coaptation. It was evaluated in the TriBAND study (*n* = 61) and showed a sustained reduction of TR (69% grade II or lower), reduction in annular diameter, inferior vena cava diameter and early evidence of right heart remodeling 1 month after implantation (69).
3) Heterotopic caval valve implantation: this approach aims to reduce retrograde cava flow and hepato-renal congestion in patients with unacceptable surgical risk who are refractory to medical treatment and unsuitable for T-TEER with initially promising results (70).
4) Transcatheter orthotopic valve implantation: this involves replacing the TV with a transcatheter valve. Preliminary data of the TRISCEND trial has been published regarding the EVOQUE system (Edwards Lifesciences, Irvine, CA, USA), a transcatheter valve that replaces the TV:TR was reduced to mild or less in 98% of the patients and there was a significant improvement in symptoms 30 days after the procedure (71).

While device therapy is rapidly evolving, more randomized trials are needed to allow us to select the right population and optimal timing for each device.

## 7. Conclusions and future directions

TR is highly prevalent, particularly when associated with left heart disease and among vulnerable populations such as women and the elderly. Although it has been historically overlooked, TR is associated with a 65% increase in hospitalizations and death, a risk that is even higher in patients with RV dysfunction. Diagnosis is made by TTE (2D and 3D). RV assessment by TTE is more challenging in the presence of severe TR.

**Figure 1:**
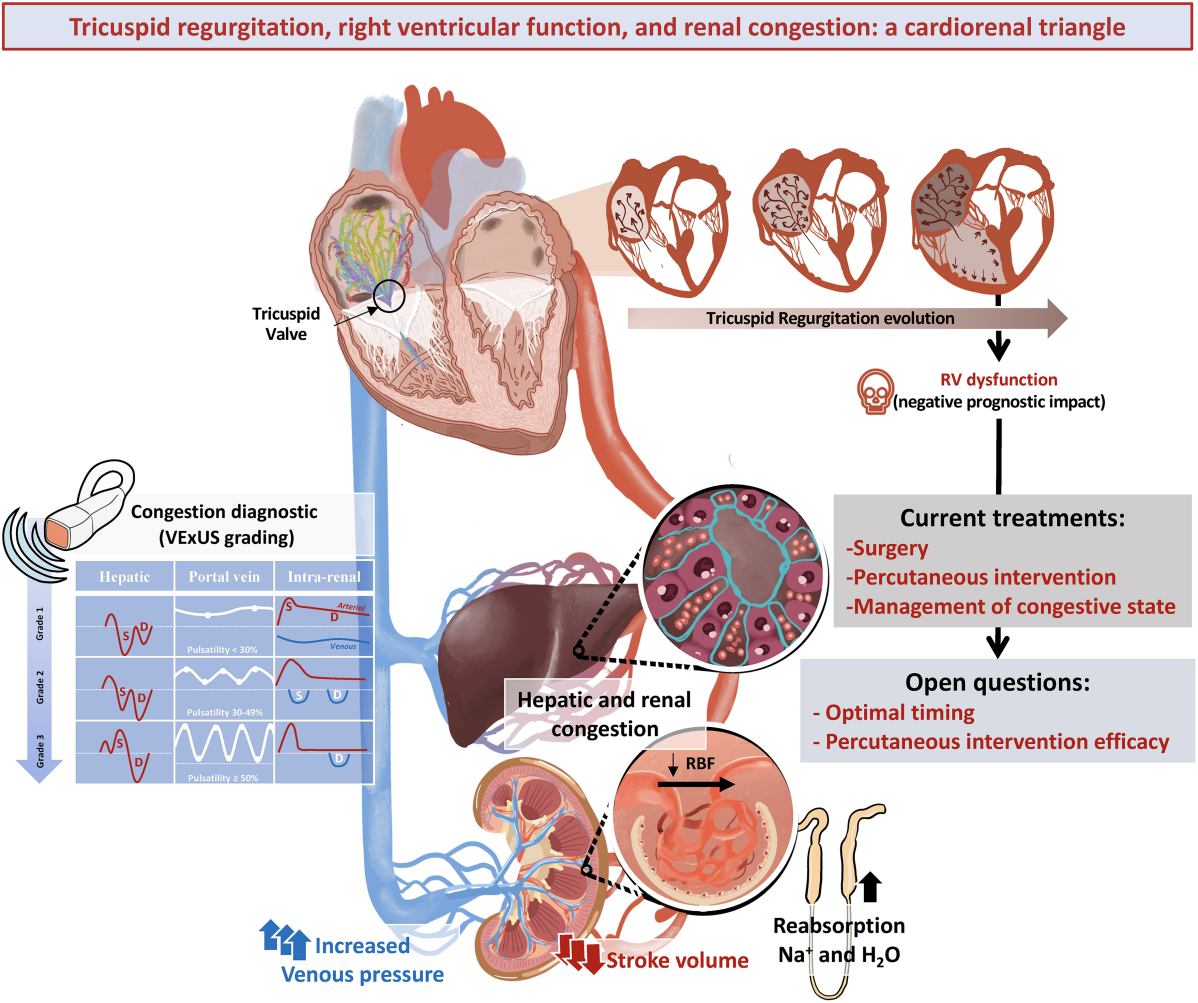
Pathophysiology, congestion diagnosis and treatment of tricuspid regurgitation. As tricuspid regurgitation progresses, it leads to right ventricular dilatation and dysfunction, worsening the prognosis. In addition, increased venous pressure and reduced stroke volume, cause congestion and affect hepatic and renal haemodynamics. Hence, a valuable tool for assessing congestion is the VExUS ultrasound protocol, which measures inferior vena cava diameter and portal, hepatic and intra-renal Doppler patterns. Treatment includes management of congestive state and tricuspid valve intervention, where percutaneous options are emerging. Key questions remain regarding the optimal timing and efficacy of percutaneous treatments. VExUS, Venous Ultrasound Score Excess; RBF, renal blood flow; RV, right ventricle; Na, sodium, H_2_O.

Clinical manifestations of TR revolve around signs of abdominal congestion, which bear prognostic implications even when mild (72). A comprehensive approach to diagnose and manage congestion includes abdominal ultrasound and biomarkers in addition to clinical evaluation. Renal, hepatic, and intestinal involvement are key findings that may perpetuate a cycle of refractory congestion and hemodynamic abnormalities. Treatment strategies are based on combination of diuretics and renal replacement techniques in refractory patients. RV failure often coexists with severe TR and represents a significant prognostic factor although there is no evidence-based medical treatment available (Figure 1).

In the recent years, several percutaneous treatment techniques have emerged, with different mechanistic approaches, showing promising safety profiles. However, there remains limited evidence regarding their clinical and prognostic impact, and more importantly their appropriate indications. More advanced patients may benefit less from the therapy, therefore identifying optimal timing for these interventions becomes a crucial element (73). Further refinement of clinical, laboratory and echocardiographic assessment are needed to establish ideal timing and preferred technique for percutaneous TR interventions. Additionally, understanding how these procedures can improve RV function combined with medical therapy to enhance decongestive effects and protect against end-organ failure is an important area for future research. This is an exciting time as emerging TR innovations hold promise to improve outcomes and quality of life in patients facing this challenging yet common condition.

## References

[1] Topilsky Y Maltais S Medina Inojosa J Oguz D Michelena H Maalouf J Burden of tricuspid regurgitation in patients diagnosed in the community setting. JACC Cardiovasc Imaging. (2019) 12(3):433–42. 10.1016/j.jcmg.2018.06.014 30121261

[2] Cahill TJ Prothero A Wilson J Kennedy A Brubert J Masters M Community prevalence, mechanisms and outcome of mitral or tricuspid regurgitation. Heart. (2021) 107(12):1003–9. 10.1136/heartjnl-2020-318482 33674352

[3] Maeder MT Holst DP Kaye DM. Tricuspid regurgitation contributes to renal dysfunction in patients with heart failure. J Card Fail. (2008) 14(10):824–30. 10.1016/j.cardfail.2008.07.236 19041045

[4] Benfari G Antoine C Miller WL Thapa P Topilsky Y Rossi A Excess mortality associated with functional tricuspid regurgitation complicating heart failure with reduced ejection fraction. Circulation. (2019) 140(3):196–206. 10.1161/CIRCULATIONAHA.118.038946 31117814

[5] Butcher SC Fortuni F Dietz MF Prihadi EA van der Bijl P Ajmone Marsan N Renal function in patients with significant tricuspid regurgitation: pathophysiological mechanisms and prognostic implications. J Intern Med. (2021) 290(3):715–27. 10.1111/joim.13312 34114700 PMC8453518

[6] Ronco C McCullough P Anker SD Anand I Aspromonte N Bagshaw SM Cardio-renal syndromes: report from the consensus conference of the acute dialysis quality initiative. Eur Heart J. (2010) 31(6):703–11. 10.1093/eurheartj/ehp507 20037146 PMC2838681

[7] Nath J Foster E Heidenreich PA. Impact of tricuspid regurgitation on long-term survival. J Am Coll Cardiol. (2004) 43(3):405–9. 10.1016/j.jacc.2003.09.036 15013122

[8] Kodali S Hahn RT Eleid MF Kipperman R Smith R Lim DS Feasibility study of the transcatheter valve repair system for severe tricuspid regurgitation. J Am Coll Cardiol. (2021) 77(4):345–56. 10.1016/j.jacc.2020.11.047 33509390

[9] Lin G Nishimura RA Connolly HM Dearani JA Sundt TM Hayes DL. Severe symptomatic tricuspid valve regurgitation due to permanent pacemaker or implantable cardioverter-defibrillator leads. J Am Coll Cardiol. (2005) 45(10):1672–5. 10.1016/j.jacc.2005.02.037 15893186

[10] Hahn RT. State-of-the-art review of echocardiographic imaging in the evaluation and treatment of functional tricuspid regurgitation. Circ Cardiovasc Imaging. [Internet]. (2016) 9(12):e005332. 10.1161/circimaging.116.005332 27974407

[11] Höke U Auger D Thijssen J Wolterbeek R Van Der Velde ET Holman ER Significant lead-induced tricuspid regurgitation is associated with poor prognosis at long-term follow-up. Heart. (2014) 100(12):960–8. 10.1136/heartjnl-2013-304673 24449717

[12] Kazum SS Sagie A Shochat T Ben-Gal T Bental T Kornowski R Prevalence, echocardiographic correlations, and clinical outcome of tricuspid regurgitation in patients with significant left ventricular dysfunction. Am J Med. (2019) 132(1):81–7. 10.1016/j.amjmed.2018.10.004 30367857

[13] Dreyfus J Ghalem N Garbarz E Cimadevilla C Nataf P Vahanian A Timing of referral of patients with severe isolated tricuspid valve regurgitation to surgeons (from a French nationwide database). Am J Cardiol. (2018) 122(2):323–6. 10.1016/j.amjcard.2018.04.003 29747858

[14] Zoghbi WA Adams D Bonow RO Enriquez-Sarano M Foster E Grayburn PA Recommendations for noninvasive evaluation of native valvular regurgitation: a report from the American society of echocardiography developed in collaboration with the society for cardiovascular magnetic resonance. J Am Soc Echocardiogr. (2017) 30(4):303–71. 10.1016/j.echo.2017.01.007 28314623

[15] Rana BS Robinson S Francis R Toshner M Swaans MJ Agarwal S Tricuspid regurgitation and the right ventricle in risk stratification and timing of intervention. Echo Res Pract. (2019) 6(1):R25–39. 10.1530/erp-18-0051 30763278 PMC6410762

[16] Vinciguerra M Sitges M Pomar JL Romiti S Domenech-Ximenos B D’Abramo M Functional tricuspid regurgitation: behind the scenes of a long-time neglected disease. Front Cardiovasc Med. (2022) 9:226. 10.3389/fcvm.2022.836441 PMC8899114 35265685

[17] Dietz MF Prihadi EA Van Der Bijl P Goedemans L Mertens BJA Gursoy E Prognostic implications of right ventricular remodeling and function in patients with significant secondary tricuspid regurgitation. Circulation. (2019) 140(10):836–45. 10.1161/CIRCULATIONAHA.119.039630 31185724

[18] Florescu DR Muraru D Florescu C Volpato V Caravita S Perger E Right heart chambers geometry and function in patients with the atrial and the ventricular phenotypes of functional tricuspid regurgitation. Eur Heart J Cardiovasc Imaging. (2022) 23(7):930–40. 10.1093/ehjci/jeab211 34747460

[19] Condello F Gitto M Stefanini GG. Etiology, epidemiology, pathophysiology and management of tricuspid regurgitation: an overview. Rev Cardiovasc Med. (2021) 22(4):1115–42. 10.31083/j.rcm2204122 34957757

[20] Prihadi EA Delgado V Leon MB Enriquez-Sarano M Topilsky Y Bax JJ. Morphologic types of tricuspid regurgitation: characteristics and prognostic implications. JACC Cardiovasc Imaging. (2019) 12(3):491–9. 10.1016/j.jcmg.2018.09.027 30846123

[21] Topilsky Y Khanna A Le Tourneau T Park S Michelena H Suri R Clinical context and mechanism of functional tricuspid regurgitation in patients with and without pulmonary hypertension. Circ Cardiovasc Imaging. (2012) 5(3):314–23. 10.1161/CIRCIMAGING.111.967919 22447806

[22] Hahn RT. Tricuspid regurgitation. N Engl J Med. (2023) 388(20):1876–91. 10.1056/NEJMra2216709 37195943

[23] Galloo X Dietz MF Fortuni F Prihadi EA Cosyns B Delgado V Prognostic implications of atrial vs. ventricular functional tricuspid regurgitation. Eur Heart J Cardiovasc Imaging. (2023) 24(6):733–41. 10.1093/ehjci/jead016 36762683 PMC10437306

[24] Schwartz LA Rozenbaum Z Ghantous E Kramarz J Biner S Ghermezi M Impact of right ventricular dysfunction and tricuspid regurgitation on outcomes in patients undergoing transcatheter aortic valve replacement. J Am Soc Echocardiogr. (2017) 30(1):36–46. 10.1016/j.echo.2016.08.016 27742242

[25] Rudski LG Lai WW Afilalo J Hua L Handschumacher MD Chandrasekaran K Guidelines for the echocardiographic assessment of the right heart in adults: a report from the American society of echocardiography. J Am Soc Echocardiogr. (2010) 23(7):685–713. 10.1016/j.echo.2010.05.010 20620859

[26] Ancona F Melillo F Calvo F Attalla El Halabieh N Stella S Capogrosso C Right ventricular systolic function in severe tricuspid regurgitation: prognostic relevance of longitudinal strain. Eur Heart J Cardiovasc Imaging. (2021) 22(8):868–75. 10.1093/ehjci/jeab030 33623973

[27] L’Official G Vely M Kosmala W Galli E Guerin A Chen E Isolated functional tricuspid regurgitation: how to define patients at risk for event? ESC Heart Fail. (2023) 10(3):1605–14. 10.1002/ehf2.14189 36811285 PMC10192508

[28] Fortuni F Butcher SC Dietz MF van der Bijl P Prihadi EA De Ferrari GM Right ventricular–pulmonary arterial coupling in secondary tricuspid regurgitation. Am J Cardiol. (2021) 148:138–45. 10.1016/j.amjcard.2021.02.037 33667451

[29] Buechel ERV Mertens LL. Imaging the right heart: the use of integrated multimodality imaging. Eur Heart J. (2012) 33(8):949–60. 10.1093/eurheartj/ehr490 22408035

[30] Khalique OK Cavalcante JL Shah D Guta AC Zhan Y Piazza N Multimodality imaging of the tricuspid valve and right heart anatomy. JACC Cardiovasc Imaging. (2019) 12(3):516–31. 10.1016/j.jcmg.2019.01.006 30846125

[31] Hahn RT Thomas JD Khalique OK Cavalcante JL Praz F Zoghbi WA. Imaging assessment of tricuspid regurgitation severity. JACC Cardiovasc Imaging. (2019) 12(3):469–90. 10.1016/j.jcmg.2018.07.033 30846122

[32] Besler C Unterhuber M Rommel KP Unger E Hartung P von Roeder M Nutritional status in tricuspid regurgitation: implications of transcatheter repair. Eur J Heart Fail. (2020) 22(10):1826–36. 10.1002/ejhf.1752 32100930

[33] Unterhuber M Kresoja K Besler C Rommel K Orban M Roeder M Cardiac output states in patients with severe functional tricuspid regurgitation: impact on treatment success and prognosis. Eur J Heart Fail. (2021) 23(10):1784–94. 10.1002/ejhf.2307 34272792

[34] Damman K Navis G Voors AA Asselbergs FW Smilde TDJ Cleland JGF Worsening renal function and prognosis in heart failure: systematic review and meta-analysis. J Card Fail. (2007) 13(8):599–608. 10.1016/j.cardfail.2007.04.008 17923350

[35] Agricola E Marini C Stella S Monello A Fisicaro A Tufaro V Effects of functional tricuspid regurgitation on renal function and long-term prognosis in patients with heart failure. J Cardiovasc Med. (2017) 18(2):60–8. 10.2459/JCM.0000000000000312 26258726

[36] Mullens W Verbrugge FH Nijst P Tang WHW. Renal sodium avidity in heart failure: from pathophysiology to treatment strategies. Eur Heart J. (2017) 38(24):1872–82. 10.1093/eurheartj/ehx035 28329085

[37] Xanthopoulos A Starling RC Kitai T Triposkiadis F. Heart failure and liver disease. JACC Heart Fail. (2019) 7(2):87–97. 10.1016/j.jchf.2018.10.007 30553904

[38] Wells ML Venkatesh SK. Congestive hepatopathy. Abdom Radiol. (2018) 43(8):2037–51. 10.1007/s00261-017-1387-x 29147765

[39] Fortea JI Puente Á Cuadrado A Huelin P Pellón R González Sánchez FJ Congestive hepatopathy. Int J Mol Sci. (2020) 21(24):9420. 10.3390/ijms21249420 33321947 PMC7764741

[40] Polsinelli VB Marteau L Shah SJ. The role of splanchnic congestion and the intestinal microenvironment in the pathogenesis of advanced heart failure. Curr Opin Support Palliat Care. (2019) 13(1):24–30. 10.1097/SPC.0000000000000414 30640740 PMC6366455

[41] Rola P Miralles-Aguiar F Argaiz E Beaubien-Souligny W Haycock K Karimov T Clinical applications of the venous excess ultrasound (VExUS) score: conceptual review and case series. Ultrasound J. (2021) 13(1):32. 10.1186/s13089-021-00232-8 34146184 PMC8214649

[42] Argaiz ER. Vexus nexus: bedside assessment of venous congestion. Adv Chronic Kidney Dis. (2021) 28(3):252–61. 10.1053/j.ackd.2021.03.004 34906310

[43] Pennestrí F Loperfido F Salvatori MP Mongiardo R Ferrazza A Guccione P Assessment of tricuspid regurgitation by pulsed Doppler ultrasonography of the hepatic veins. Am J Cardiol. (1984) 54(3):363–8. 10.1016/0002-9149(84)90198-X 6465017

[44] Iranpour P Lall C Houshyar R Helmy M Yang A Choi JI Altered Doppler flow patterns in cirrhosis patients: an overview. Ultrasonography. (2016) 35(1):3–12. 10.14366/usg.15020 26169079 PMC4701371

[45] Soler M Miñana G Santas E Núñez E de la Espriella R Valero E CA125 outperforms NT-proBNP in acute heart failure with severe tricuspid regurgitation. Int J Cardiol. (2020) 308:54–9. 10.1016/j.ijcard.2020.03.027 32209267

[46] Núñez J Espriella R Miñana G Santas E Llácer P Núñez E Antigen carbohydrate 125 as a biomarker in heart failure: a narrative review. Eur J Heart Fail. (2021) 23(9):1445–57. 10.1002/ejhf.2295 34241936

[47] Núñez J Bayés-Genís A Revuelta-López E ter Maaten JM Miñana G Barallat J Clinical role of CA125 in worsening heart failure: a BIOSTAT-CHF study subanalysis. JACC Heart Fail. (2020) 8(5):386–97. 10.1016/j.jchf.2019.12.005 32171764

[48] Goetze JP Balling L Deis T Struck J Bergmann A Gustafsson F. Bioactive adrenomedullin in plasma is associated with biventricular filling pressures in patients with advanced heart failure. Eur J Heart Fail. (2021) 23(3):489–91. 10.1002/ejhf.1937 32558059

[49] Zilinski JL Shah RV Gaggin HK Lou GM Wang TJ Januzzi JL. Measurement of multiple biomarkers in advanced stage heart failure patients treated with pulmonary artery catheter guided therapy. Crit Care. (2012) 16(4):R135. 10.1186/cc11440 22830581 PMC3580720

[50] deFilippi C Daniels LB Bayes-Genis A. Structural heart disease and ST2: cross-sectional and longitudinal associations with echocardiography. Am J Cardiol. (2015) 115(7):59B–63B. 10.1016/j.amjcard.2015.01.042 25702279

[51] Boorsma EM ter Maaten JM Damman K van Essen BJ Zannad F van Veldhuisen DJ Albuminuria as a marker of systemic congestion in patients with heart failure. Eur Heart J. (2023) 44(5):368–80. 10.1093/eurheartj/ehac528 36148485 PMC9890244

[52] Caravaca Pérez P Nuche J Morán Fernández L Lora D Blázquez-Bermejo Z López-Azor JC Potential role of natriuretic response to furosemide stress test during acute heart failure. Circ Heart Fail. (2021) 14(6):633–43. 10.1161/CIRCHEARTFAILURE.120.008166 34129364

[53] Vahanian A Beyersdorf F Praz F Milojevic M Baldus S Bauersachs J 2021 ESC/EACTS guidelines for the management of valvular heart disease: developed by the task force for the management of valvular heart disease of the European society of cardiology (ESC) and the European association for cardio-thoracic surgery (EACTS). Rev Esp Cardiol. (2022) 75(6):524. 10.1016/j.recesp.2021.11.023 35636831

[54] Rodríguez-Espinosa D Guzman-Bofarull J De La Fuente-Mancera JC Maduell F Broseta JJ Farrero M. Multimodal strategies for the diagnosis and management of refractory congestion. An integrated cardiorenal approach. Front Physiol. (2022) 13:1–18. 10.3389/fphys.2022.913580 PMC9304751 35874534

[55] Grossekettler L Schmack B Brockmann C Wanninger R Kreusser MM Frankenstein L Benefits of peritoneal ultrafiltration in HFpEF and HFrEF patients. BMC Nephrol. (2020) 21(1):179. 10.1186/s12882-020-01777-x 32410664 PMC7222460

[56] Misra M Vonesh E Van Stone JC Moore HL Prowant B Nolph KD. Effect of cause and time of dropout on the residual GFR: a comparative analysis of the decline of GFR on dialysis. Kidney Int. (2001) 59(2):754–63. 10.1046/j.1523-1755.2001.059002754.x 11168959

[57] François K Ronco C Bargman JM. Peritoneal dialysis for chronic congestive heart failure. Blood Purif. (2015) 40(1):45–52. 10.1159/000430084 26111872

[58] Khanin Y. Peritoneal dialysis. In: Hughes GJ, editor. A medication guide to internal medicine tests and procedures. 1st ed. Elsevier (2022). p. 217–20.

[59] Jefferies HJ Virk B Schiller B Moran J McIntyre CW. Frequent hemodialysis schedules are associated with reduced levels of dialysis-induced cardiac injury (myocardial stunning). Clin J Am Soc Nephrol. (2011) 6(6):1326–32. 10.2215/CJN.05200610 21597028 PMC3109928

[60] Chertow GM Levin NW Beck GJ Depner TA Eggers PW Gassman JJ In-center hemodialysis six times per week versus three times per week. N Engl J Med. (2010) 363(24):2287–300. 10.1056/NEJMoa1001593 21091062 PMC3042140

[61] Lim P Sharifi J Huguet R Gallet R Aouate D. Repetitive use of levosimendan in severe functional tricuspid regurgitation. Eur Heart J Acute Cardiovasc Care. (2023) 12(5):336–7. 10.1093/ehjacc/zuad013 36989396

[62] Alajaji W Baydoun A Al-Kindi SG Henry L Hanna MA Oliveira GH. Digoxin therapy for cor pulmonale: a systematic review. Int J Cardiol. (2016) 223:320–4. 10.1016/j.ijcard.2016.08.018 27543702

[63] Lam PH Keramida K Filippatos GS Gupta N Faselis C Deedwania P Right ventricular ejection fraction and beta-blocker effect in heart failure with reduced ejection fraction. J Card Fail. (2022) 28(1):65–70. 10.1016/j.cardfail.2021.07.026 34419597

[64] Palau P Seller J Domínguez E Sastre C Ramón JM de La Espriella R Effect of β-blocker withdrawal on functional capacity in heart failure and preserved ejection fraction. J Am Coll Cardiol. (2021) 78(21):2042–56. 10.1016/j.jacc.2021.08.073 34794685

[65] Taramasso M Benfari G van der Bijl P Alessandrini H Attinger-Toller A Biasco L Transcatheter versus medical treatment of patients with symptomatic severe tricuspid regurgitation. J Am Coll Cardiol. (2019) 74(24):2998–3008. 10.1016/j.jacc.2019.09.028 31568868

[66] Karam N Braun D Mehr M Orban M Stocker TJ Deseive S Impact of transcatheter tricuspid valve repair for severe tricuspid regurgitation on kidney and liver function. JACC Cardiovasc Interv. (2019) 12(15):1413–20. 10.1016/j.jcin.2019.04.018 31126888

[67] Orban M Braun D Deseive S Stolz L Stocker TJ Stark K Transcatheter edge-to-edge repair for tricuspid regurgitation is associated with right ventricular reverse remodeling in patients with right-sided heart failure. JACC Cardiovasc Imaging. (2019) 12(3):559–60. 10.1016/j.jcmg.2018.10.029 30660521

[68] Sorajja P Whisenant B Hamid N Naik H Makkar R Tadros P Transcatheter repair for patients with tricuspid regurgitation. N Engl J Med. (2023) 388(20):1833–42. 10.1056/NEJMoa2300525 36876753

[69] Nickenig G Friedrichs K Baldus S Arnold M Seidler T Hakmi S Thirty-day outcomes of the cardioband tricuspid system for patients with symptomatic functional tricuspid regurgitation: the TriBAND study. EuroIntervention. (2021) 17(10):809–17. 10.4244/EIJ-D-21-00300 34031021 PMC9724867

[70] Estévez-Loureiro R Sánchez-Recalde A Amat-Santos IJ Cruz-González I Baz JA Pascual I 6-Month outcomes of the tricvalve system in patients with tricuspid regurgitation: the TRICUS EURO study. JACC Cardiovasc Interv. (2022) 15(13):1366–77. 10.1016/j.jcin.2022.05.022 35583363

[71] Kodali S Hahn RT George I Davidson CJ Narang A Zahr F Transfemoral tricuspid valve replacement in patients with tricuspid regurgitation: TRISCEND study 30-day results. JACC Cardiovasc Interv. (2022) 15(5):471–80. 10.1016/j.jcin.2022.01.016 35272771

[72] González-Gómez A Fernández-Golfín C Hinojar R Monteagudo JM García A García-Sebastián C The 4A classification for patients with tricuspid regurgitation. Revista Española de Cardiología (English Edition). (2023) S1885-5857 (23) 00068-3. 10.1016/j.rec.2023.02.008. 36898521

[73] Lara-Breitinger KM Scott CG Nkomo VT Pellikka PA Kane GC Chaliki HP Tricuspid regurgitation impact on outcomes (TRIO): a simple clinical risk score. Mayo Clin Proc. (2022) 97(8):1449–61. 10.1016/j.mayocp.2022.05.015 35933133

